# The Dutch Healthy Diet index (DHD-index): an instrument to measure adherence to the Dutch Guidelines for a Healthy Diet

**DOI:** 10.1186/1475-2891-11-49

**Published:** 2012-07-20

**Authors:** Linde van Lee, Anouk Geelen, EvelineJCHooft van Huysduynen, Jeanne H M de Vries, Pieter van’t Veer, Edith J M Feskens

**Affiliations:** 1 Division of human Nutrition, Wageningen University, 8129, 6700 EV, Wageningen, The Netherlands

**Keywords:** The Netherlands, Index, Dietary patterns, Dietary guidelines

## Abstract

**Background:**

The objective was to develop an index based on the Dutch Guidelines for a healthy Diet of 2006 that reflects dietary quality and to apply it to the Dutch National Food Consumption Survey (DNFCS) to examine the associations with micronutrient intakes.

**Methods:**

A total of 749 men and women, aged 19–30 years, contributed two 24-hour recalls and additional questionnaires in the DNFCS of 2003. The Dutch Healthy Diet index (DHD-index) includes ten components representing the ten Dutch Guidelines for a Healthy Diet. Per component the score ranges between zero and ten, resulting in a total score between zero (no adherence) and 100 (complete adherence).

**Results:**

The mean ± SD of the DHD-index was 60.4 ± 11.5 for women and 57.8 ± 10.8 for men (P for difference = 0.002). Each component score increased across the sex-specific quintiles of the DHD-index. An inverse association was observed between the sex-specific quintiles of the DHD-index and total energy intake. Calcium, riboflavin, and vitamin E intake decreased with increasing DHD-index, an inverse association which disappeared after energy adjustment. Vitamin C showed a positive association across quintiles, also when adjusted for energy. For folate, iron, magnesium, potassium, thiamin, and vitamin B6 a positive association emerged after adjustment for energy.

**Conclusions:**

The DHD-index is capable of ranking participants according to their adherence to the Dutch Guidelines for a Healthy Diet by reflecting variation in nine out of ten components that constitute the index when based on two 24-hour recalls. Furthermore, the index showed to be a good measure of nutrient density of diets.

## Introduction

Diets have a complex nature, as foods and nutrients are consumed in combinations which can induce interactions and synergies between dietary components. Dietary pattern analysis, therefore, is assumed a more appropriate approach for investigating diet-disease associations than focusing on a single food or nutrient [1-4].

One approach of assessing dietary patterns is to construct an a priori dietary index. These indices are mainly based on national or international dietary recommendations, which are designed to decrease the risk of chronic diseases and nutrient deficiencies [5,6]. Indices can be used to measure dietary quality in populations and monitor it over time [7] or measure changes in diets in intervention studies [8]. Furthermore, in epidemiological studies an index can be used to investigate the diet-disease associations [9]. Additionally, confounding by diet can be controlled through the use of a dietary pattern variable or a diet index score [10]. A well-known example of an index is the American Healthy Eating Index-2005 (HEI-2005) [11,12]. This index has been associated with, health outcomes [13], and has been used as monitoring tool in American populations [7]. However, the HEI-2005 cannot be used for the Dutch situation, because the American dietary guidelines are different from those in the Netherlands. The 2005 Dietary guidelines for Americans, on which the HEI-2005 is based, mention all the major food groups while the Dutch guidelines do not. Furthermore, the Dutch guidelines include a restriction on the number of consumption occasions with acidic drinks and foods (ADF) [5].

To date, two Dutch indices have been developed by Löwik et al. [14], both based on the Dutch Guidelines for a Healthy Diet of 1986. The first dietary quality index consisted of five criteria: less than 35% energy from total fat, less than 10% energy from saturated fatty acids (SFA), less than 33 mg/MJ cholesterol, more than 50% energy from carbohydrates and less than 25% energy from mono- and disaccharides. For each criterion, one point was assigned to individuals who adhered. The score, ranging from zero (low quality) to five (high quality), was inversely related to energy intake and positively associated with a higher prevalence of following a prescribed diet and a higher educational level [14]. The second index was a food-based dietary guideline index with seven components. The score, ranging from zero (low quality) to seven (high quality), was positively associated with energy intake and all evaluated nutrient densities (calcium, iron, vitamin A, thiamin, riboflavin, vitamin B6 and vitamin C) [14].

In 2006, the Dutch Guidelines for a Healthy Diet were revised by the health Council of the Netherlands by adding new guidelines on physical activity, number of consumption occasions with ADF and excluding the guidelines on cholesterol and mono- and disaccharides[5]. Furthermore, evidence-based quantitative recommendations for vegetable, fruit, fish, trans fatty acids (TFA), and alcohol consumption were formulated. The guideline for ADF is added to the guidelines in view of the prevention of dental caries and risk reduction of dental erosion. Due to the revision of the Dutch guidelines, no Dutch index is yet available. Therefore, we developed a new index, the Dutch Healthy Diet index (DHD-index), based on the Dutch Guidelines for a Healthy Diet of 2006 [5], the official background document [15] and the information provided by the Netherland Nutrition Centre (NNC) [16]. Furthermore, we applied the index to data of the Dutch National Food Consumption Survey of 2003 (DNFCS-2003) to examine the associations with micronutrient intakes. We hypothesized that participants with higher DHD-index scores will have both higher intakes of vitamins and minerals and have a more nutrient-dense diet.

## Materials and Methods

The DNFCS-2003 is a population-wide food consumption survey in the Netherlands and has been described in detail elsewhere [17]. Briefly, data were collected in 2003 and respondents (n = 750) were men and women aged 19–30 years and randomly selected from a representative consumer panel of households. One participant was excluded from these analyses due to an incomplete SQUASH, which led to a total of 749 participants. Their dietary intake was assessed by two non-consecutive 24-hour recalls administered by telephone using Epic-Soft. EPIC-Soft is a computerized 24-hour recall program that follows standardized steps [18,19]. Recall days were randomly selected from all days of the week. Characteristics of the recall days such as following a diet regime and special day were asked during the 24-hour recalls. In addition, a baseline questionnaire was administered on subjects’ characteristics (weight, height, age, education, and income) and demographics (postal code), and the short questionnaire to assess health enhancing physical activity (SQUASH) was administered. Furthermore, a food frequency questionnaire (FFQ) was included to assess consumption frequencies of episodically consumed foods (e.g. fish, eggs, chips). After data collection, macronutrient and micronutrient intakes were estimated by using the Dutch food composition database of 2001[20]. We selected the micronutrients calcium, folate, iron, magnesium, potassium, riboflavin, thiamin, vitamin A, vitamin B6, vitamin B12, vitamin C and vitamin E by relevance and availability in the database [16,21]. Furthermore, a quality check was done on inconsistencies between first and second interview on general data as birth date. Differences in energy ratio between interviewers and weeks of data collection were checked by using the estimated energy intake divided by estimated basal metabolic rate. Missing values, false answers (that were not in range of possible answers) and typing errors were changed in EPIC soft using the original recall data. Underreporting, based on the estimated energy intake divided by estimated basal metabolic rate, was observed to be 11%.

### Development of the DHD-index

The DHD-index is a continuous score with ten components that represent the ten Dutch Guidelines for a Healthy Diet of 2006 (Table 1). By choosing a continuous scoring system we assume that we can observe changes in diets of intervention studies better than with a dichotomous scoring system. For all components a maximum of ten points can be allotted, resulting in a range of zero to 100 points. The components physical activity, vegetable, fruit, fish, and fiber are adequacy components and the components SFA, TFA, number of consumption occasions of ADF, sodium and alcohol are moderation components. Cut-off values represent the required amount of consumption or physical activities undertaken (minimum for adequacy and maximum for moderation components), whereas the threshold values represents the level of intake that deserves zero points for the moderation components. For the component ADF the threshold value was lower than the recommended maximum of seven ADF consumption occasions. Consequently, this component was scored dichotomously. The components and their cut-off and threshold values are shown in Table 1.

**Table 1:** Components and Dutch dietary guidelines of the DHD-index and their cut-off (maximum score) and threshold values (minimum score)

	**Components**	**Dutch Guidelines for a Healthy Diet**	**Minimum score (=0)**	**Maximum score (=10)**
1.	Physical activity (week)	At least 30 minutes of moderate intensity physical activity – brisk walking, cycling, gardening, etc. – at least five days a week, but preferably every day.	0 activities^*^	≥ 5 activities
2.	Vegetable (day)	Eat 150 to 200 grams of vegetables.	0 gram	≥200 gram
3.	Fruit + fruit juices (day)^*^	Eat 200 grams of fruit a day.	0 gram	≥ 200 gram
4.	Fiber (day)	Eat 30 to 40 grams a day of dietary fiber, especially from sources such as fruit, vegetables and whole-grain cereal products.	0 gram/4.2 MJ	≥14 gram/4.2 MJ
5.	Fish (day) ^†^	Eat two portions of fish a week, at least one of which should be oily fish.	0 mg EPA + DHA	≥ 450 mg EPA + DHA
6.	SFA (day)	Limit saturated fatty acid consumption to less than 10 percent of energy intake.	≥ 16.6 en%	< 10 en%
7.	TFA (day)	Limit mono trans-fatty acid consumption to less than 1 percent of energy intake.	≥ 1.6 en%	< 1 en%
8.	ADF (day)^‡^	Limit consumption of foods and beverages that contain easily fermentable sugars and drinks that are high in food acids, to seven occasions a day (including main meals).	> 7 occasions	≤ 7 occasions
9.	Sodium (day)	Limit consumption of table salt to 6 grams a day.	≥ 2.45 gram	< 1.68 gram
10.	Alcohol (day)	If alcohol is consumed at all, male intake should be limited to two Dutch units (20 gram ethanol) a day and female intake to one.	Male: ≥60 grams Female: ≥40 grams	Male: ≤20 grams Female: ≤10 grams

SFA = saturated fatty acids, TFA = trans fatty acids, ADF = acidic drinks and foods.

^*^maximum of 100 gram of juice could be included.

^†^EPA and DHA intake from foods and fish oil capsules.

^‡^the number of consumption occasions was defined as the number of hours where at least one food or drink with a pH<5.5 and total acidity>0.5% was consumed.

The first component assesses physical activity; the Health Council of the Netherlands recommends being active for minimally 30 minutes of at least moderate intensity for at least five days per week [5]. The second component is based on the recommendation of 150–200 grams of vegetables per day. The higher of the two recommendations was chosen as the cut-off value of the component. The third component is based on the recommendation of 200 grams of fruits per day. The NNC communicates that a maximum of 100 grams can be replaced by fruit juices which naturally contain folate and vitamin C [22]. In the DNFCS-2003 six types of juice complied with the criterion (orange juice with and without pulp, pineapple juice, berry juice, grapefruit juice and mixed fruit juice) and could be included in the fruit group for a maximum of 100 grams in total. The fourth component is based on the recommendation of 30–40 grams of dietary fiber per day. The criterion used was stated in the background document and was 14 grams dietary fiber per 4.2mJ per day [15]. The fifth component, fish, is estimated based on the fish fatty acids eicosapentaenoic acid (EPA) and docosahexaenoic acid (DHA), which are likely to be the protective components of fish [15]. At least 450 mg/day of these fish fatty acids are recommended [15] and their intake can be achieved by fish consumption or by using fish oil capsules. Although, fish consumption is preferred by the Health Council of the Netherlands, fish oil capsules are permitted as substitute for fish for people who do not eat fish [23]. Fish oil capsules were assumed to contain 200 mg of fish fatty acids per capsule, based on labeling information of the fish oil capsules available in the Netherlands. The average daily intake of EPA and DHA from the capsules was added to the 2-day average intake of EPA and DHA from fish. The sixth and seventh components were based on the recommendations to consume less than 10 energy percent of SFA and less than one energy percent of TFA respectively. The eighth component is based on the maximum recommended number of ADF consumption occasions which is seven occasions per day including the three main meals. The operational definition of a ADF consumption occasion is every half an hour where a food item or drink with a pH level lower than 5.5 and a total acidity higher than 0.5% is consumed [24]. Consumption of less than 2.4 grams of sodium per day, as recommended in the corresponding guideline, is scored in component nine. In the DNFCS and most other studies, no data is available on salt added during cooking and at the table. The contribution of sodium of these sources was assumed to be on average about 30 % of total sodium intake, based on available literature [25-27]. Therefore, we lowered the cut-off and threshold value for this component by 30%. The last component, alcohol, is differentiated by sex. For men, the recommendation is to consume maximally two Dutch units of alcohol, and for women to consume maximally one Dutch unit per day. One Dutch unit of alcohol contains 10 grams of ethanol [5].

### Scoring

All scores were based on the 2-days average intake. For the adequacy components, the minimum score of zero was allotted when there was no consumption, or no activity. The scores for the intakes or activities between zero and the cut-off value were calculated by dividing the reported intake or activity, by the cut-off value and subsequently multiplying this ratio by ten. The maximum score of ten points was allotted if the recommended amount of intake, or activities, was achieved.

For the moderation components, we determined threshold values above which to assign the score of zero, because no scientific evidence specifies the quantity of intake that deserves zero points. The threshold values were determined based on the 85^th^ percentiles of the 2-day average intakes of the sample population. For alcohol intake, however, evidence on upper levels is available and we used the criteria for binge drinking as threshold value [28]. Zero points were allotted when reported intakes were above the threshold values. Ten points were allotted when intake were below the cut-off values. The scores for intake between threshold and cut-off value were calculated by dividing the difference between the intake and cut-off value by the difference between threshold and cut-off value, and subsequently multiplying this ratio by ten. Because the score has to decrease when intake increases, the outcome was subtracted from ten. The ADF component was scored dichotomously and only 3.5% of the population was assigned a score of zero, while all others received a score of ten.

To be able to apply the DHD-index to the data of the DNFCS-2003, two components were adapted due to limitations of the dataset. Firstly, the SQUASH reported activities per week and not per day. Ten points were allotted when five activities per week, meeting the recommendation, were reported. It was not known on how many days these activities were performed. Secondly, the component ADF was redefined as the number of hours during which foods or drinks fulfilling the criterion were consumed, because intake data was available per hour.

### Data analysis

All food and nutrient intakes and number of ADF occasions were averaged over two days before being used to score individual dietary intakes. Sex-specific quintiles of the DHD-index scores were estimated. Means across the quintiles were tested using P for trend from linear regression analysis. Micronutrient intake was reported with and without total energy adjustment. Adjusted intakes are presented as mean nutrient intakes per 9.8mJ, which was the average energy intake of the population. For the component fruit, a sensitivity analysis was done by excluding the fruit juices. SAS (version 9.2, SAS Institute, Cary, NC) was used for all calculations and a P value of <0.05 was considered statistically significant.

## Results

Mean ± SD age of the population was 25.0 ± 3.6 years and did not differ between women and men. BMI was significantly higher for women (24.5 ± 4.6) compared to men (23.3 ± 3.2), as was prevalence of supplement use (17.5% vs. 9.6% respectively) and following a diet regime (9.9% vs. 0.9% respectively). Furthermore, 26.5% of women were classified as lower educated compared to 18.5% of men. The distribution of recalls over week and weekend days did not differ between men and women. The mean ± SD DHD-index score for the total population was 59.2 ± 11.2 and it was significantly higher for women than for men (mean difference of 2.4 points; Table 2). Women scored significantly higher on the components physical activity, dietary fiber, sodium and alcohol, whereas men scored significantly higher on the components vegetable, SFA and TFA. No significant differences between men and women were observed for the components fruit, fish and ADF.

**Table 2:** Mean (SD) scores of the DHD-index components in 749 Dutch men and women aged 19–30 years

	**Total (n = 749)**	**Men (n = 352)**	**Women (n = 397)**	P-value between sex^*^
DHD-index	59.2 (11.2)	57.8 (10.8)	60.4 (11.5)	0.002
1. Physical activity	9.4 (1.9)	9.1 (2.3)	9.7 (1.4)	0.001
2. Vegetable	4.8 (2.9)	5.2 (2.9)	4.4 (2.8)	<0.001
3. Fruit	4.6 (3.7)	4.4 (3.7)	4.8 (3.6)	0.130
4. Fiber	6.1 (2.3)	5.9 (2.3)	6.3 (2.3)	0.022
5. Fish	1.1 (2.4)	1.1 (2.4)	1.1 (2.3)	0.798
6. SFA	5.2 (3.5)	5.5 (3.4)	4.9 (3.5)	0.011
7. TFA	7.0 (3.9)	7.5 (3.6)	6.5 (4.0)	0.005
8. ADF	9.7 (1.8)	9.6 (2.0)	9.7 (1.6)	0.267
9. Sodium	2.4 (3.8)	1.1 (2.6)	3.6 (4.2)	<0.001
10. Alcohol	8.9 (2.8)	8.4 (3.3)	9.3 (2.1)	<0.001

^*^independent *t*-test comparing men and women.

SFA = saturated fat, TFA = trans fatty acids, ADF = acidic drinks and foods.

The DHD-index score was normally distributed and ranged from 28.1 to 88.0 in men and from 24.4 to 95.0 in women. All components within the DHD-index showed a significant positive association across the sex-specific quintiles of the index (Table 3). Energy intake was inversely associated with the DHD-index (P < 0.001; Table 4). Following a diet regime, prescribed or on own initiative, was positively associated with the DHD-index score (P = 0.005). Age, BMI, education and prevalence of supplement use did not show a significant trend across quintiles of the index score.

**Table 3:** Distribution of components scores (means (SD)) across sex-specific quintiles of the DHD-index in 749 Dutch men and women^*^

	**Sex-specific quintiles of Dutch Healthy Diet index**					
	**1 (n = 148)**	**2 (n = 150)**	**3 (n = 151)**	**4 (n = 149)**	**5 (n = 150)**	**P for trend**
DHD-index	43.8 (5.2)	52.9 (2.2)	58.7 (2.1)	65.2 (2.6)	75.0 (4.9)	<0.001
1. Physical activity	8.7 (2.8)	9.5 (1.6)	9.5 (1.6)	9.5 (1.9)	9.8 (1.0)	<0.001
2. Vegetable	3.5 (2.6)	4.3 (2.5)	4.6 (2.8)	5.1 (2.9)	6.6 (2.8)	<0.001
3. Fruit	2.1 (2.5)	3.4 (3.1)	3.9 (3.5)	5.7 (3.5)	7.8 (2.9)	<0.001
4. Fiber	4.5 (1.8)	5.0 (1.7)	6.1 (2.0)	6.7 (2.2)	8.1 (1.9)	<0.001
5. Fish	0.6 (1.4)	0.8 (1.8)	0.8 (1.8)	1.3 (2.6)	2.0 (3.4)	<0.001
6. SFA	3.3 (3.6)	3.9 (3.3)	4.8 (3.1)	6.1 (3.0)	7.6 (2.6)	<0.001
7. TFA	3.8 (4.0)	5.8 (4.0)	7.7 (3.5)	8.3 (2.8)	9.3 (2.1)	<0.001
8. ADF	8.9 (3.1)	9.8 (1.4)	9.9 (1.1)	9.7 (1.6)	9.9 (0.8)	<0.001
9. Sodium	0.7 (2.1)	1.8 (3.3)	2.3 (4.2)	3.3 (4.2)	4.0 (4.3)	<0.001
10. Alcohol	7.7 (3.7)	8.5 (3.2)	9.0 (2.6)	9.4 (1.9)	9.8 (0.9)	<0.001

*cut-off quintiles men: 47.7, 54.9, 60.6, 67.2.

cut-off quintiles women: 50.4, 56.5, 62.8, 70.6.

SFA = saturated fat, TFA = trans fatty acids, ADF = acidic drinks and foods.

**Table 4:** Distribution of characteristics (means (SD)) across sex-specific quintiles of the DHD-index in 749 Dutch men and women*

	**Sex-specific quintiles of Dutch Healthy Diet index**					
	**1 (n = 148)**	**2 (n = 150)**	**3 (n = 151)**	**4 (n = 149)**	**5 (n = 150)**	**P for trend**
Age (y)	24.8 (3.7)	25.1 (3.5)	24.8 (3.5)	24.7 (3.5)	25.4 (3.7)	0.346
BMI (kg/m^2^)	23.9 (3.9)	24.1 (4.2)	23.6 (4.2)	24.1 (3.9)	24.0 (3.9)	0.799
Energy intake (MJ/day)	11.1 (3.5)	10.4 (3.2)	9.8 (3.0)	9.3 (3.0)	8.3 (2.7)	<0.001
Supplements (%)	28.2	20.8	32.5	24.7	29.3	0.750
Diet regime^†^ (%)	2.0	5.4	10.6	4.6	12.0	0.005
Education^‡^ (%)						0.059
Low	25.7	28.0	23.8	20.8	15.2	
Moderate	44.6	46.7	44.4	55.0	50.1	
High	29.7	25.3	31.8	24.2	33.8	

*cut-off quintiles men: 47.7, 54.9, 60.6, 67.2.

cut-off quintiles women: 50.4, 56.5, 62.7, 70.6.

^†^Diet regime: Salt restriction, fat/cholesterol restriction, diabetes, energy restricted, energy restricted (own initiative), light digestible, lactose restricted, vegetarian (no meat/fish), antroposophical, other.

^‡^low education=primary school, vocational and lower general secondary education. Moderate=higher secondary education and intermediate vocational training. High=higher vocational education and university.

For the micronutrients calcium, and vitamin E significant inverse associations across sex specific quintiles of the DHD-index scores were observed (Table 5). However, when these intakes were adjusted for mean energy intake these associations disappeared. Riboflavin also showed an inverse association across quintiles of the DHD-index, however, after adjustment for energy intake the association changed to a positive association. For the micronutrients folate, iron, magnesium, potassium, thiamin, and vitamin B6, significant positive associations with the DHD-index score were shown for the energy adjusted intakes, but not for the unadjusted intakes. Vitamin C was positively associated across the quintiles both in mg/day and in mg/9.8mJ.

**Table 5:** Means (SD) of selected micronutrients across sex-specific quintiles of DHD-index in 749 Dutch men and women^*^

	** Sex-specific quintiles of Dutch healthy diet index**			
**Micronutrients (day)**	**1 (n = 148)**	**3 (n = 151)**	**5 (n = 150)**	**P for trend**
Calcium (mg)	1744 (880)	1632 (719)	1414 (693)	<0.001
Folate (mcg)	169 (76)	166 (92)	177 (91)	0.344
Iron (mg)	9.3 (3.1)	9.8 (5.7)	9.1 (3.8)	0.999
Magnesium (mg)	309 (126)	299 (125)	290 (116)	0.257
Potassium (mg)	3056 (1129)	2991 (1080)	3036 (1096)	0.975
Riboflavin (mg)	1.5 (0.)	1.5 (0.7)	1.3 (0.6)	0.002
Thiamin (mg)	1.0 (0.4)	1.0 (0.4)	1.0 (0.5)	0.119
Vitamin A (RE)^†^	974 (882)	975 (1031)	905 (775)	0.420
Vitamin B6 (mg)	1.6 (0.8)	1.5 (0.7)	1.6 (0.7)	0.857
Vitamin B12 (mcg)	3.8 (2.1)	3.6 (5.4)	3.2 (2.8)	0.118
Vitamin C (mg)	63 (40)	76 (48)	108 (62)	<0.001
Vitamin E (mg)	12.6 (7.0)	10.5 (5.8)	9.6 (5.0)	<0.001
Calcium (mg)	1598 (775)	1739 (902)	1786 (1086)	0.201
Folate (mcg)	149 (48)	167 (71)	211 (86)	<0.001
Iron (mg)	8.5 (2.5)	10.0 (6.8)	10.9 (3.5)	<0.001
Magnesium (mg)	272 (64)	303 (116)	345 (102)	<0.001
Potassium (mg)	2718 (673)	3039 (906)	3649 (1077)	<0.001
Riboflavin (mg)	1.4 (0.6)	1.5 (0.8)	1.5 (0.7)	0.014
Thiamin (mg)	0.9 (0.3)	1.0 (0.5)	1.2 (0.4)	<0.001
Vitamin A (RE)^†^	882 (770)	987 (1001)	1073 (894)	0.058
Vitamin B6 (mg)	1.4 (0.4)	1.5 (0.7)	1.9 (0.6)	<0.001
Vitamin B12 (mcg)	3.4 (2.1)	3.7 (4.3)	3.8 (3.2)	0.189
Vitamin C (mg)	57 (40)	79 (50)	134 (80)	<0.001
Vitamin E (mg)	11.1 (5.0)	10.3 (4.7)	11.2 (4.4)	0.952

^*^cut-off quintiles men: 47.7, 54.9, 60.6, 67.2.

cut-off quintiles women: 50.4, 56.5, 62.7, 70.6.

^†^RE = retinol equivalents.

When as part of a sensitivity analysis fruit intake was estimated excluding the intake of fruit juices, mean intake decreased by 83 grams, and the mean score changed from 4.6 to 3.7 points. In total 139 (18.6%) subjects adhered to the guideline when fruit juices were included as compared to 106 (14.2%) subjects based on whole fruit consumption only. The correlation between the scores with and without juices was very high (r=0.91, p<0.001).

## Discussion

The DHD-index is capable of ranking participants according to their adherence to the Dutch Guidelines for a Healthy Diet by reflecting variation in the components that constitute the index, except for the component ADF. This component showed a low variation and is consequently not discriminative in ranking subjects according to their adherence to the guidelines. Furthermore, the index score is positively associated with ’following a diet regime’ and inversely associated with energy intake, which were not included in the index. Additionally, the DHD-index showed to be a good measure of nutrient density of diets.

The components of the DHD-index were based on three different documents about the guidelines: the guidelines as communicated by the health council of the Netherlands [5], the background document describing the guidelines and the evidence in more detail [15] and the information provided by the NNC [16]. The NNC communicates the guidelines in a more understandable way and provides food-based examples of the dietary guidelines to the general Dutch population and subpopulations. These three documents were more or less comparable to each other and we decided to stay as close as possible to the guidelines, with three exceptions. For the component dietary fiber, the background document indicated an energy-dependent recommendation which was more specific than the range of 30–40 gram mentioned in the guidelines. For the fish component, the background document had a specified recommended amount of fish fatty acids instead of consuming two portions of fish. The third exception was the fruit component, which was based on the recommendations of the NNC [22]. The NNC communicates that 100 grams of fruit can be replaced by all fruit juices complying to the criteria of naturally containing vitamin C and folate [22]. The sensitivity analysis showed that the total scores increased by an average of 0.86 after the inclusion of fruit juices.

For the threshold values of the moderation components, the 85^th^ percentiles of the current population were used, as was done by others [11]. Although we used the 85^th^ percentiles of the 2-day average, the HEI-2005 used the 1-day distribution [11]. Also other indices, such as the heart disease prevention eating index [29] and the Mediterranean diet score [30], used the distribution of intake of the population under study for determining cut-off values. However, because of the use of the 85^th^ percentiles of the distribution of the 2-day averages of 19-30-year-olds, the results of the DHD-index cannot be compared with other Dutch subpopulations, as the cut-off values will differ. An evidence-based threshold value for all moderation components, like the binge-drinking threshold values for the alcohol component, would be the most preferred. However, for the other moderation components these do not exist. Yet, a more appropriate solution would be to use 85^th^ percentiles of usual or long-term intakes of a reference dataset representative of the total Dutch population for all future use.

All ten components of the DHD-index have similar weights, as mentioned in the guidelines [5]. However, some components were correlated, which indicates an overlap in dietary behaviors which causes indirectly more weight to that dietary behavior. The components vegetables and fruit were correlated to the dietary component fiber (r=0.36 and r=0.32, respectively), which can be explained by the fact that fiber represents consumption of vegetables and fruit in addition to wholegrain products. The correlation between the component SFA and TFA was 0.29, which is plausible as these fatty acids appear partly in the same products [15,31]. These correlations should be studied in future research to explore the effect of the additional weight on diet-disease relations. If judged necessary, differential weighting of the components could be applied.

We hypothesized that participants who adhered to a higher degree to the Dutch Guidelines for a Healthy Diet, have both higher absolute intakes of micronutrients and a more nutrient-dense diet. However, only vitamin C intake increased across quintiles of the DHD-index when energy was not taken into account. The intake of the micronutrients folate, iron, magnesium, potassium, thiamin, riboflavin and vitamin B6 only showed a positive association across quintiles of the DHD-index after adjustment for energy intake. This latter result indicates that participants in the higher quintiles of the DHD-index have a more nutrient-dense composition of the diet. However, they have a lower absolute intake of these micronutrients, because of the inverse association of energy intake across quintiles of the DHD-index. The intake of calcium, riboflavin, and vitamin E showed a decline across the quintiles. Nevertheless, the mean average intake in all quintiles was still acceptable compared to the recommended average intakes [32], which made the lower intakes less worrisome for public health practices. The inverse association of these three micronutrients disappeared after energy adjustment.

In contrast to energy intake, BMI was not inversely associated with the DHD-index score. This result may be due in part to the self-reported nature of the dietary data, which could invoke underreporting [33]. It can also be caused by specific subject characteristics like restrained eating in the higher quintiles of the DHD-index score. This hypothesis can be confirmed by the increasing percentage of participants following a diet regime in the higher quintiles of the DHD-index score. Unfortunately, no data on other subject characteristics as eating behavior or true energy intake was available in the DNFCS-2003. In the HEI-2005, energy intake from solid fats, alcoholic beverages, and added sugars is included as component of the index [11]. For the Dutch situation, no operational guideline for energy intake is available. The health council states that the guidelines are meant for the apparently healthy population with a healthy and stable weight. Consequently, no component is constructed for energy intake in the DHD-index. Energy adjustment should be therefore applied when examining diet-disease associations.

The adherence to the physical activity criterion was quite high compared to previously described physical activity levels in the Netherlands [34]. This may be due to a possible over-reporting by using the SQUASH [35], although it is a validated questionnaire for estimating usual physical activity [36]. It was suggested by Ocké et al. [17] that the population under study was slightly different compared to the general Dutch population in the same age category, which may partly explain the high level of physical activity.

The average score of the component ADF ranged from 8.9 to 9.9 across quintiles, consequently, the variation of this component was low (SD = 1.8). Therefore this component is not that discriminative in ranking subjects according to their adherence to the guidelines. The component was included in the Dutch guidelines because it is important for the prevention of teeth erosion, which is quite different from the aims for prevention of chronic diseases and nutrient deficiencies of the other recommendations [5]. We advise to adapt or delete the component ADF from the index in future research, if variation in the component appears to be low in other studies as well.

Data on sodium intake is expected to be underestimated through lacking information on sodium added at the dinner table and during cooking. We have tried to correct for this by lowering the guideline by 30%. However, the variation in intake of sodium within the population was ignored by this method, which could have biased the results. Preferably, sodium intake is measured in 24-hour urine samples, which is considered the standard for measuring sodium intake [37].

The estimation of the components of the DHD-index was based on the 2-day average of dietary intake. Although two non-consecutive 24-hour recalls are acceptable for assessing dietary intake on group level [38], the 2-day average will not be a good estimate to assess usual intake distributions for some components, e.g. fish and alcohol, due to a low frequency of consumption. A FFQ designed to assess usual intake could give better estimates for intake of episodically consumed foods. A FFQ, however, is designed for ranking participants according to their intake and not for estimation of absolute intakes [39]. Moreover, a FFQ cannot be used to estimate the component ADF. Statistical models as the Multiple-Source-Method or the National Cancer Institute method can be used to estimate usual intake distributions or individual usual intakes [40-45]. However, these statistical models have their limitations as well. Altogether, dietary assessment methods are prone to errors which will be reflected by the estimates of the DHD-index. Therefore, care should be taken when comparing DHD-index scores based on different dietary assessment methods.

## Conclusion

The DHD-index can be used to estimate the adherence to the Dutch Guidelines for a Healthy Diet and is a good measure of nutrient density of diets. In future research the DHD-index can be used as monitoring tool in public health research or as tool for assessing a Dutch dietary pattern and studying diet-health associations.

## Abbreviations

DHD-index, Dutch Healthy Diet-index; DNFCS, Dutch National Food Consumption Survey; SD, Standard Deviation; HEI-2005, Healthy Eating Index-2005; TFA, Trans Fatty Acids; NNC, Netherlands Nutrition Centre; SQUASH, Short Questionnaire to Assess health enhancing physical activity; FFQ, Food Frequency Questionnaire; SFA, Saturated Fatty Acids; ADF, Acidic Drinks and Foods; EPA, Eicosapentaenoic Acid; DHA, Docosahexaenoic Acid; BMI, Body Mass Index.

## Competing interest

The authors declare that they have no competing interest.

## Authors contributions

The contributions of the authors were as follows: LL participated in the manuscript conception, statistical analyses, data interpretation, manuscript writing and revising; AG participated in manuscript conception, data interpretation, writing and review; EH, JV and PV contributed to data interpretation and review, EF participated in manuscript conception, data interpretation, writing and review. All of the authors contributed to the critical revision of the manuscript. All authors read and approved the final manuscript.
